# Book Review: Deep Inequality: Understanding the New Normal and How to Challenge It

**DOI:** 10.3389/fsoc.2019.00004

**Published:** 2019-02-04

**Authors:** Glenn W. Muschert

**Affiliations:** Department of Humanities and Social Sciences, Khalifa University of Science and Technology, Abu Dhabi, United Arab Emirates

**Keywords:** inequality, stratificati on, neoliberahsation, new normal economy, narrative analysis

*Deep Inequality: Understanding the New Normal and How to Challenge It* is Earl Wysong and Robert Perrucci's latest book to examine trends in class relations, including the declining life chances of American workers in the last decade. The focus of *Deep Inequality* is a narrative analysis of the “new normal,” defined as a corporate media narrative that explains persistent and expanding economic inequalities in America since the Great Recession of 2008. The classic rock band *The Who* famously declared: “Meet the new boss, same as the old boss,” within the context of protestations that, we “Won't Get Fooled Again.” Unfortunately, as argued adroitly in *Deep Inequality*, it nonetheless seems that economically squeezed Americans have, for the past decade, adjusted to the seemingly inevitable “new normal,” while economic prospects, hopes for their children, job security, and retirement outlooks have declined.

Unfortunately, to the extent that Americans direct their outrage about their declining economic prospects away from the ruling economic class and their minions, they have indeed been fooled again. *Deep Inequality* clarifies the origin and development of an ideology known as the “new normal,” which is an elite-driven rhetoric of explaining and justifying economic hardships and challenges experienced in the last decade by the 80 per cent of Americans who occupy the bottom of the stratification hierarchy. Meanwhile, the top 1 per cent (the wealthiest Americans) and their minions in the next 19 per cent (executives, senior management, and highly-credentialed professionals) continue to capture nearly all of the benefits of economic growth experienced in the last decade, while the other 80 per cent (salaried professionals, wage workers, the self-employed, and the economic underclass) continues to struggle to stay afloat.

The heart of the book is a coherent explication of the rhetorical development and expansion over time of the new normal ideology. The discussion draws heavily on key statements about the new normal derived from corporate media, political discourse, and public relations organizations (whether private or public sector), all of which come to clarify that the new normal is structurally inevitable, that there is no option but to adjust, and that such economic struggle is often the result of individual failures. The explicit goal of the book, largely met by the authors, is to clarify the development of the new normal ideology as a way of justifying the *status quo* of economic relations. The authors also point out that there is indeed an alternative rhetoric in existence that contradicts the new normal ideology, which they term the “structural realities analysis.” This alternative to the dominant new normal discourse is one which views inequality as emerging from the institutional and organizational inequalities present in society as a whole, including those reflected in media, education, healthcare, and employment structures. This description of the new normal and the structural realities ideologist are the focus of Chapter 1.

In Chapter 2, the authors proceed to describe income and wealth inequality more generally, as they describe the new class structure as a double diamond with a small privileged class (20 per cent) above a larger new working class (80 per cent). After discussing the new normal and new class structure, the book moves through a number of more focused discussions of aspects of economic reality, including the new national and global economy (Chapter 3), the new reality of work and the workplace (Chapter 4), the political sphere (Chapter 5), educational institutions (Chapter 6), and in information and culture industries (Chapter 7). As long as readers are cognizant of the discussion in the first two chapters, the focused discussions in the subsequent chapters are highly accessible and readable. The eighth and final chapters bring a number of threads together, and outlines what the authors predict could be the future of inequality in America. Chapter 8 presents a description of increasing political polarization and political gridlock, in which partisan issues seem to drive all policy discussion and action, while leading to inaction due to the seeming unwillingness of parties to reach compromise. This is explored via the topic of climate change, and how the issue was handled at the end of the Obama administration and into the early months of the Trump administration. These points are solidly argued within a sociological context melding a variety of conceptual perspectives including, cultural Marxism (e.g., Althusser), framing, and social constructionism. The analysis also draws on the scholarship of Noam Chomsky, Naomi Klein, and C. Wright Mills.

The most significant accomplishment of the book is to clarify the origin and development of the new normal, and to point out that this ideology is a rhetorical tool of elites to “explain away” the economic hardships caused by structural inequalities in social institutions. The authors clearly and convincingly reassert the social in social inequality, as they argue that stratification emerges from social structural relations, not as a result of individual shortcomings or failings or abstract notions of globalization affecting economic relations. They also convincingly remind the reader that the new normal ideology is not just a construction of conservatives such as Republicans, but rather is used by Democrats and Republicans alike, as the new normal is featured in a variety of rhetorical venues such as those typically viewed as conservative as well as those considered more progressive.

Engaging this volume may lead a reader to understand the tension between the new normal and the structural realities narratives, however this cognitive dissonance remains unleveraged. The reader might wish for more discussion and clarity about how to resist the new normal both as individuals and collectively. While the title of the volume implies such discussion of how to challenge the new normal, this discussion is limited in the text to generalities. The question of “What is to be done?” remains largely unexplored in a practical sense. Admittedly, expecting a road map for displacing the new normal with a progressive alternative may be a tall order, but after reading *Deep Inequality* the reader might be ready to consider a plan for concrete action. While it is possible to see the dominance of the new normal as a reason for pessimism about the future of inequalities in America, it is worth pointing out that the structural realities narrative persists despite the vast imbalance of funding in favor of those promulgating the new normal. A case in point is that the structural realities approach exists and persists, despite the fact that corporate media, private public relations, and politicians backed by lobbying groups generally express their views of inequalities via the new normal narrative.

Wysong and Perrucci's *Deep Inequality* is very readable, and contributes to the contemporary discussion of economic inequalities and the ideologies used to justify them. It will be suitable, in whole or in part, as a supplementary text for undergraduate and postgraduate courses in social stratification. It is also accessible to the general educated reader seeking a critical view of contemporary economic inequalities. *Deep Inequality* provides a solidly sociological view of the current status and ideology of inequality in contemporary America. The final section of the book calls for the creation of a progressive “new, new normal,” yet to be articulated, but which would be an antidote to the *status quo*. Indeed this book clarifies the dominant contemporary rhetoric justifying the *status quo*, and armed with this knowledge, the reader can engage in a search for such new ideologies and practical solutions.

## Author Contributions

The author confirms being the sole contributor of this work and has approved it for publication.

### Conflict of Interest Statement

The author declares that the research was conducted in the absence of any commercial or financial relationships that could be construed as a potential conflict of interest.

